# A monocenter, patient-blinded, randomized, parallel-group, non-inferiority study to compare cochlear implant receiver/stimulator device fixation techniques (COMFIT) with and without drilling in adults eligible for primary cochlear implantation

**DOI:** 10.1186/s13063-023-07568-7

**Published:** 2023-09-24

**Authors:** Laura M. Markodimitraki, Timen C. ten Harkel, Edwin Bennink, Inge Stegeman, Hans G. X. M. Thomeer

**Affiliations:** 1 Department of Otorhinolaryngology and Head & Neck Surgery, University Medical Center Utrecht, G.05.1.29, P.O. Box 85500, 3508 GA Utrecht, the Netherlands; 2 UMC Utrecht Brain Center, Utrecht University, Utrecht, the Netherlands; 3 Department of Oral and Maxillofacial Surgery, University Medical Center Utrecht, Utrecht, the Netherlands; 4 Image Sciences Institute, University Medical Center Utrecht, Utrecht University, Utrecht, The Netherlands

**Keywords:** Cochlear implant, Fixation technique, Bony well, Tight subperiosteal pocket, Otology, RCT, Migration, CBCT

## Abstract

**Background:**

During the cochlear implantation procedure, the receiver/stimulator (R/S) part of the implant is fixated to prevent postoperative device migration, which could have an adverse effect on the position of the electrode array in the cochlea. We aim to compare the migration rates of two fixation techniques, the bony recess versus the subperiosteal tight pocket without bony sutures.

**Methods and analysis:**

This single-blind randomized controlled trial will recruit a total of 112 primary cochlear implantation adult patients, eligible for implantation according to the current standard of practice. Randomization will be performed by an electronic data capture system Castor EDC, with participants block randomized to either bony recess or standard subperiosteal tight pocket in a 1:1 ratio, stratified by age. The primary outcome of this study is the R/S device migration rate; secondary outcomes include patient-experienced burden using the validated COMPASS questionnaire, electrode migration rate, electrode impedance values, speech perception scores, correlation between R/S migration, electrode array migration and patient complaints, assessment of complication rates, and validation of an implant position measurement method. Data will be collected at baseline, 1 week, 4 weeks, 8 weeks, 3 months, and 12 months after surgery. All data analyses will be conducted according to the intention-to-treat principle.

**Discussion:**

Cochlear implantation by means of creating a tight subperiosteal pocket without drilling a bony seat is a minimally invasive fixation technique with many advantages. However, the safety of this technique has not yet been proven with certainty. This is the first randomized controlled trial that directly compares the minimally invasive technique with the conventional method of drilling a bony seat.

**Trial registration:**

Netherlands Trial Register NL9698. Registered on 31 August 2021.

**Supplementary Information:**

The online version contains supplementary material available at 10.1186/s13063-023-07568-7.

## Background

### Background and rationale

Cochlear implants (CIs) provide hearing through direct electrical stimulation of the auditory nerve in patients with sensorineural hearing loss and have become standard care for adult and pediatric patients with severe to profound bilateral hearing loss. Cochlear implantation surgery requires careful planning and execution. The correct electrode array positioning in the cochlea is crucial for optimal functionality of the device. This array is connected to the body of the implant, known as the receiver/stimulator (R/S) device. During cochlear implantation, the R/S device is placed and fixated on the skull. It should be placed close to the pinna, without possible interference of the microphone in the behind-the-ear device laying (partially) on top of the R/S device.

The standard fixation technique of the R/S device, which is recommended by the surgeon’s guide that is supplied with the respective implants, consists of drilling out a part of the bony cortex of the skull (a bony recess), as well as non-absorbable suture retaining holes [1]. The bony recess technique lowers the profile of the R/S device in relation to the skull surface and holds it in place with non-absorbable sutures to the bone. Although rare, complications that are due to drilling of the bony recess can have serious consequences. Depending on the extent of drilling and the cortex thickness of the patient, the dura mater is sometimes exposed [2, 3]. Several studies report on dural tears with subsequent cerebrospinal fluid leakage as a direct result of extensive cortical drilling [4–6]. Other complications that have been reported (but occur very rarely) and associated with this technique are late-onset hematomas, epi-/subdural hematoma, tentorial herniation, and cerebral infarction, as well as meningitis [5, 7–11]. To avoid such risks, in recent years ENT surgeons have adopted less invasive techniques [1–3, 10, 12–20]. Additionally, later CI models have a lower profile and a flatter bottom. However, the lowering of the profile is a trade-off for a larger footprint which results in a larger bony recess thus a larger area of the skull is drilled out.

Complications that can occur as a result of failed fixation of the R/S is a shift/migration of the internal components of the implant: the R/S device itself and the electrode array [16, 21]. Migration of the R/S device can lead to pain/headache, behind-the-ear device problems, hematoma, device failure, and in some cases necessitating revision surgery [19, 22–26]. It can also have an effect on the position of the electrode array in the cochlea. Electrode migration or extrusion is one of the most common indications for revision surgery [23–25]. This complication can cause poor performance, pain, vertigo, tinnitus, and facial nerve stimulation, but can also present without complaints [27]. Increase in impedance values has also been described as a result of electrode array migration [28]. The rate of reported electrode migration varies in the literature and seems to occur more than previously thought [27, 29].

A minimally invasive technique that does not require drilling out a bony recess, known as the subperiosteal tight pocket technique, was first described by Balkany et al. in 2009 [10]. This technique uses the anatomical boundaries of the pericranium to create a tight subperiosteal pocket in which the R/S device is inserted. Apart from the advantage of not having to drill out a bony recess, thus eliminating the risk of complications associated with the bony recess, the subperiosteal tight pocket technique also has the advantage of a smaller incision and shorter operating time [16]. Creating the subperiosteal pocket might also require less manipulation and straining of the temporalis muscle (compared to the mentioned bony recess technique), thereby reducing postoperative pain or tissue-related complaints even more.

Since the publication of the study by Balkany et al. in 2009, many ENT specialists are applying the tight subperiosteal technique [1, 2, 12–14, 16–18, 20, 30–33]. However, since the R/S device is not fixated in a bony recess or by sutures, migration of the device is a point of concern due to the complications that can occur. To evaluate the difference in migration rates between the fixation technique currently used in our center (the bony recess technique), and the intervention technique (subperiosteal tight pocket technique), we conducted a literature review [34]. The results were inconclusive due to a lack of high-quality studies from a methodological point of view. Thus, there is no quality evidence to support the superiority of either technique. Therefore, in the COMFIT trial, we aim to compare the subperiosteal tight pocket technique with the bony recess technique, for fixation of the R/S device of the cochlear implant.

#### Objectives

The primary objective of our study is to compare the migration rates of the two fixation techniques (bony recess vs. subperiosteal tight pocket) by analyzing 3D reconstructions of the R/S device, acquired by cone-beam computed tomography (CBCT) scans at baseline and at 3 and 12 months post-surgery. Secondary objectives are to investigate the difference between the two fixation techniques in patient-experienced burden using the validated COMPASS questionnaire, electrode array migration rate, and electrode impedance values. Other secondary objectives are to investigate the association of electrode impedance values with R/S device and electrode migration, and whether complaints of performance drop, vertigo, tinnitus, headache, or nonauditory stimulation are associated with electrode array migration and R/S device migration. We will also compare the complication rate of these surgical techniques, for major and minor complications. Finally, we will validate the measurement method technique with flexible tape measure for the assessment of migration of the R/S device [35].

#### Trial design

**Fig. 1:**
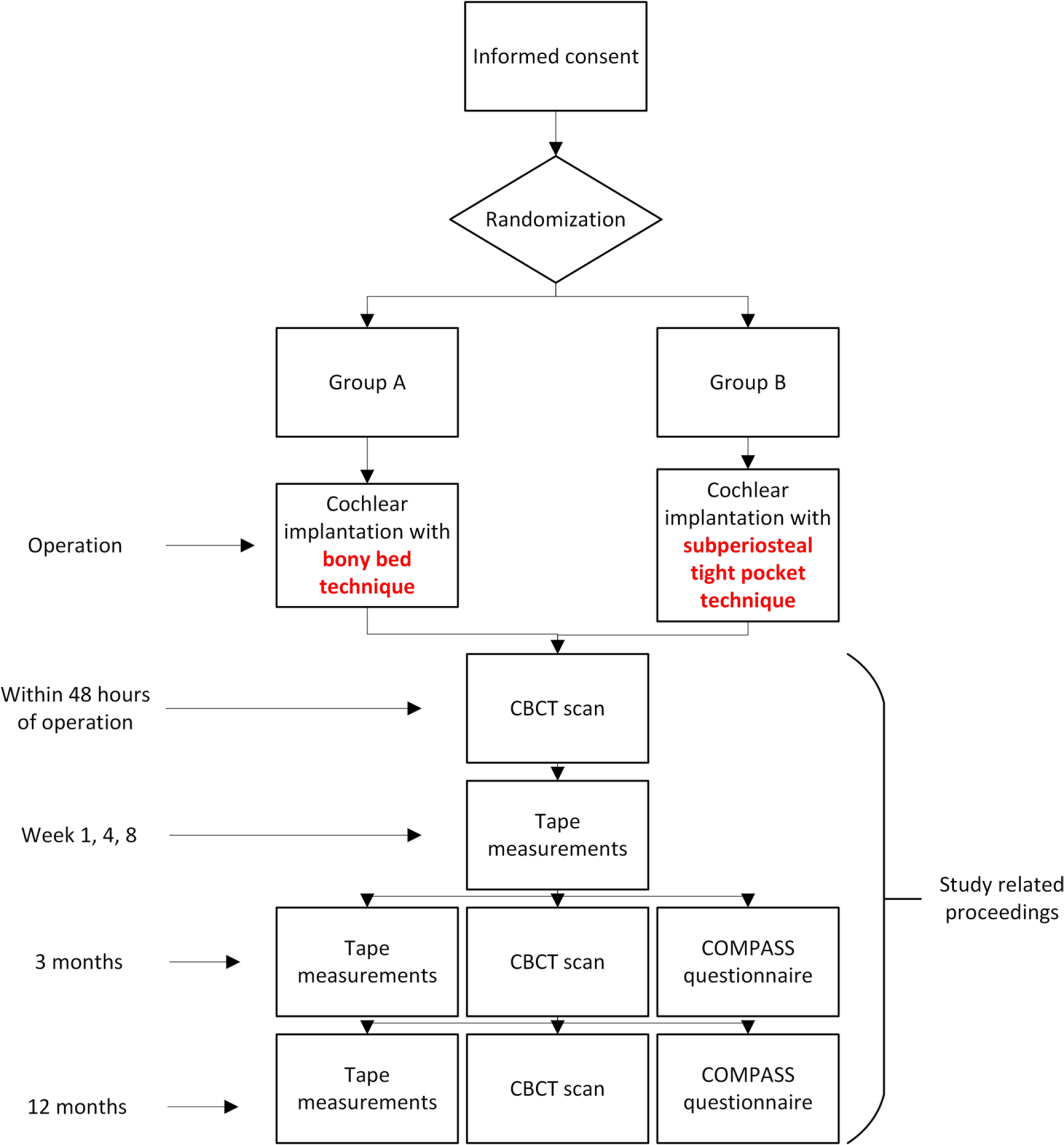
Flowchart of the study. CBCT: cone-beam CT scan; COMPASS questionnaire: patient-reported outcome measure on cochlear implant awareness. Patients will be randomized into two groups according to variable, weighted block randomization module subgroups with stratification for age (18–50 years, and > 50 years)

This is a single-blind, non-inferiority randomized controlled trial, with two study arms (Fig. 1). Patients will be randomly allocated into equally sized groups: group A and group B (allocation ratio 1:1). Patients in group A will be operated with the bony recess technique, and patients in group B will be operated with the subperiosteal tight pocket technique. Inclusion in the study will have no consequence for the model or brand chosen by the patient, as is currently standard practice.

## Methods: participants, interventions, and outcomes

### Study setting

This is a monocenter study performed in a tertiary referral clinic in the Netherlands, the University Medical Center (UMC) Utrecht.

### Eligibility criteria

The study population consists of adult patients (> 18 years old) that are approved for cochlear implantation according to standard care criteria. Patients will initially undergo a series of diagnostic tests to assess eligibility for cochlear implantation. These are as follows: a CT scan, a pure tone audiogram/speech test, psychological evaluation, and a consultation by the audiologist and ENT specialist. The Cochlear Implant Team of the UMC Utrecht will assess the work-up results and assess eligibility for cochlear implantation, according to the current clinically applied criteria.

All cochlear implant models will be included in this study. The choice for the cochlear implant model lies with the patient and the CI team of the UMC Utrecht and will not be affected by taking part in this trial. In order to be eligible to participate in this study, a participant must have provided written informed consent authorization before participating in the study. They also must have Dutch written language proficiency and be physically able to undergo a CBCT scan.

A potential participant who is a revision or reimplantation candidate, is unable to understand or sign informed consent, or is pregnant during the trial will be excluded from participation in this study.

### Interventions: description

The standard surgical procedures for cochlear implantation will be followed. A retroauricular S-shaped incision will be made to expose the mastoid. The electrode array will be inserted via a posterior tympanotomy and round window implantation by soft-surgery techniques. The R/S device will be fixated according to the group the patient is allocated to. The bony recess technique will be used in group A; a bony recess will be drilled at an angle of 30 to 60° relative from the Frankfurt Horizontal plane. The provided silicone dummy will be used to ensure the depth and dimensions of the recess are sufficient. No tie-down sutures will be used. Patients allocated to group B will be operated using the subperiosteal tight pocket technique as described by Balkany et al. [10].

### Interventions: modifications

Modifying the allocated intervention would require a revision surgery where the cochlear implant would be removed. Revision surgery is potentially harmful for the patient; therefore, it will only be performed in rare cases such as device failure, wound infection, or persisting pain.

### Interventions: adherence

The measurement scans will be performed on the same days as the regular follow-up visits of the medical rehabilitation program.

### Interventions: concomitant care

Not applicable, this study does not alter the regular care pathway.

### Outcomes

**Fig. 2:**
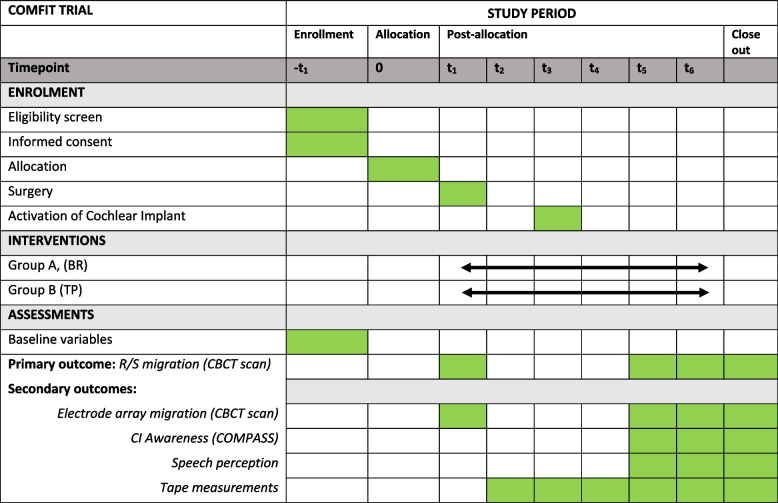
Schedule of enrolment, interventions, and assessments adapted from the Standard Protocol Items: Recommendations for Interventional Trials (SPIRIT). −t1: 2 weeks before surgery. t1: within 48 h of cochlear implantation. t2: 1 week post-surgery. t3: 4 weeks post-surgery. t4: 8 weeks post-surgery. t5: 3 months post-surgery. t6: 12 months post-surgery

At intake, demographic data will be extracted from the electronic patient database: age, gender, if the deafness is pre- or postlingual, and electronic address. The following outcomes will be assessed at the baseline visit and follow-up visits at 1, 4, and 8 weeks and at 3 and 12 months postoperatively (Fig. 2). All measurements will be performed by the research team following the same protocol procedures.

#### Primary outcome measure

The main outcome of this study is R/S device migration and will be calculated by analyzing 3D reconstructions of the R/S device, acquired by cone-beam CT (CBCT) scans at baseline and during follow-up and at 12 months. We consider migration either translational or rotational above 1.0 mm or 1° as clinically relevant. Any migration under these cut-offs is considered within the measurement error margin of the analysis (0.3 mm or degrees). These calculations will be carried out by using 3DMedX® (v1.2.24.1, 3D Lab Radboudumc, Nijmegen). R/S positions will be superimposed, compared, and analyzed based on the Iterative Closest Point (ICP) algorithm [36]. The 3D reconstructions of the R/S device at baseline and 12 months will be compared to calculate the primary outcome measure.

#### Secondary outcome measures

##### Electrode migration

Electrode array migration is defined as a displacement of the basal electrode outside the cochlea of ≥ 1 mm (i.e., approx. 1 contact spacing). To compare the electrode array migration rate between the two surgical techniques, we will analyze the acquired CBCT scan images following a previously validated method [29, 37]. Electrodes, situated at the level of the round window, will be categorized as extracochlear, since electrodes at this position do not provide adequate stimulation or accurate pitch perception so that they mostly have to be removed from the stimulation map.

##### Electrode impedance values

Electrode impedance values will be measured by the patient’s audiologist at 1, 3, and 12 months after operation for all electrodes. This is according to regular care. Electrode impedance values measured in kOhm (a measure of the resistance to current flow) in common ground stimulation. Values above 20–30 kOhms will be considered abnormally high. Additionally, an increase of impedance values ≥75% from the averaged baseline after 1 month of activation will be considered a significant increase [29, 38].

##### Speech perception

Three months and 1 year after cochlear implantation, a conventional speech perception test with/without noise test will be performed with CVC words from the “Nederlandse Vereniging voor Audiologie” (NVA) word-list. Each list contains 11 words with a total of 33 phonemes. This is according to regular care. The speech tests can be quantified with a simple correct percentage score in bimodal solution if the patient has a hearing aid in the contralateral ear.

##### Complications

Incidence and degree of complications according to the following categories:

- Device failure, which is classified into hard or soft failure using standardized criteria described in the 2005 Cochlear Implant Soft Failures Consensus Development Conference Statement [39].
- Major and minor complications according to the proposal of Hansen et al. [40].
  - ◦Major: a significant medical problem (e.g., meningitis), additional major surgery (e.g., cholesteatoma surgery or reimplantation due to a patient-related problem), explantation of the device for any reason other than device-related failure, any degree of permanent disability (e.g., facial nerve paralysis)
  - ◦Minor: complications leading to extended hospitalization or treatment on an outpatient basis, complications settling spontaneously or by conservative medical treatment, and complications managed by a minor surgical procedure (e.g., simple hematoma aspiration by syringe).

##### Questionnaire

The questionnaire used in this study is the validated COchlear iMPlant AwareneSS (COMPASS) survey that assesses patient-experienced burden by wearing the CI in their day-to-day activities [41]. It contains 15 items, multiple choice as well as visual analog scale items. Each item had a maximum score of 5, with a total maximum score of 75. A higher COMPASS score represents a higher awareness level. This questionnaire was developed in Utrecht and validated for use in the Dutch language. The questionnaire will be sent at 3 and 12 months postoperatively, by email through the data capture system Castor EDC to the study participants. If a patient does not wish to fill out the questionnaire online, it will be sent by post.

##### Validation of measurement method

The measurement technique to determine the position of the R/S device using a flexible tape measure, previously validated [35], will be compared to the results of migration measured by the CBCT scans. Repeated measurements will be done, and results will be compared to the results of migration measured by the CBCT scans.

### Participant timeline

All patients will undergo a high-resolution CBCT within 48 h after surgery, to assess the R/S device and electrode array position. Patients will undergo two more CBCT scans at 3 and 12 months postoperatively. The R/S device position will also be assessed with a validated external measurement method after 1, 4, and 8 weeks, and at 3 and 12 months postoperatively by a researcher or by the patients’ audiologist or speech therapist [35]. Patients will fill in a questionnaire after 3 and 12 months postoperatively. See Fig. 1 for an overview.

### Sample size

This sample size calculation was based on the primary outcome, R/S migration after 12 months. Due to limited quality evidence on the migration rate for both techniques, an estimation of the migration rates cannot be based on literature; therefore, we base our assumptions on clinical expertise [34, 42, 43]. We consider a migration under 1.0 mm or 1° to be clinically irrelevant. Migration under these cut-off points is within the measurement error margin of the measurement technique of the CBCT scan analysis. A sample size of 51 per study arm reaches 80% power (*β* = 0.8) and a significance level (*α*) of 0.05 with a non-inferiority margin of 1.0 mm. Standard deviation is estimated at 2.0 mm based on the database of Maxwell et al. [43]. In order to cover for possible loss to follow-up estimated at a maximum of 10%, we will include 56 patients per study arm.

### Recruitment

Patient recruitment started in October 2021; we anticipate recruiting approximately 60 patients per year; thus, recruitment should be completed in 2 years. Patients are recruited from the outpatient Otorhinolaryngology department at the University Medical Center Utrecht. Eligible patients will be informed about the study by their treating physician. These patients have already been approved for cochlear implant surgery by the CI team. The investigator provides the patient with an information letter and informed consent form, which is signed by both the investigator and the patient before the surgery. Patients consent to the use of their data for the research purposes outlined in this protocol which includes publication of the results once the trial has been completed. Patients will not receive compensation for participation in the trial.

### Randomization and blinding

Patients will be randomly assigned to one of the two study groups with 56 patients allocated in each group. Randomization takes place in the UMC Utrecht-endorsed electronic data capture system Castor EDC. (https://www.castoredc.com/). After giving informed consent, patients will be randomized with an allocation ratio of 1:1 and variable block sizes, with stratification for age (18–50 years, >50 years). Stratification is applied in both study groups. This is a single-blind study, meaning that only participants are blinded for the treatment allocation. The randomization will be done before surgery and patients will not be informed about the allocation. The research team is not blinded. The outcome data will be blindly analyzed. Blinding of the data will be performed by the electronic Case Report Form system used (see section “Data collection plan”). In the event that a revision surgery is necessary for removal or repositioning of the CI, unblinding is permitted. A member of the research team will inform the subject of the allocation.

## Methods: data collection, management, and analysis

### Data collection plan

After giving informed consent, the patient will receive a unique identifier, after which members of the research team will extract all necessary clinical parameters from the electronic health records (EHRs, HiX) into an electronic Case Report Form (eCRF) the UMCU endorsed system Castor EDC. Castor EDC is a browser-based, metadata-driven EDC software solution and workflow methodology for building and managing online databases. The eCRF contains data items as specified in this research protocol. Modification of the eCRF will be made only if deemed necessary and in accordance with an amendment to the research protocol. Access to the eCRF is password protected and specific roles are assigned (e.g., study coordinator, investigator, monitor). The assessors are specialized in the field of otology and are therefore trained to interpret the results of the various outcome measures (CBCT scans, speech performance tests, impedance values). Participant retention will be promoted by efficient schedule strategies, namely inviting participants for follow-up appointments on the same days as the clinical rehabilitation consults. The study participants will receive a separate invitation for each follow-up appointment, shortly before.

### Data management

Data handling and protection are conducted according to the ISO 27001-compliant processes and ICH-GCP and applicable regulations. Confidentiality will be maintained at all times and participant information will not be disclosed to third parties. After giving informed consent, the patient will receive a unique identifier. All generated (meta)data will be stored in a secure research folder structure for access control. Only researchers directly involved in the study and the monitor of the study are allowed to access the key-linking table to enable patient re-identification. The paper data files and informed consent will be stored in a locked cabin in a locked room. Only research members directly involved in this study and the monitor of the study will get access to all of the collected research data. When required, authorized personnel of the study can access the pseudonymized source data for intermediate analysis or business intelligence reports.

### Statistical analysis

To assess whether continuous variables are normally distributed, histograms and Q-Q plots will be computed. Continues data will be expressed as mean ± standard deviation (SD) when normally distributed, and as median ± interquartile range (IQR) when skewed. Number of cases and percentages will be presented as categorical variables. A *p*-value <0.05 is considered statistically significant. All analyses will be conducted according to the intention-to-treat principle.

#### Statistical analysis primary objective

The main outcome is R/S device migration calculated by analyzing 3D reconstructions of the R/S device, acquired by CBCT scans at baseline and during follow-up. Migration will be reported in millimeters and angle degrees (continuous variables) between the intervention group and the control group at baseline and 3 and 12 months after implantation. Differences between the intervention and control group will be calculated using the unpaired *t*-test or the Mann-Whitney *U* test.

#### Statistical analysis secondary objectives

COMPASS questionnaire scores between intervention and control at 3 and 12 months after cochlear implantation will be calculated using the unpaired *t*-test (or the Mann-Whitney *U* test).

Statistical analysis of electrode migration data will be compared in a number of cases and percentages. To calculate any association between electrode migration of ≥ 1 mm and R/S device migration, and between electrode migration and a decrease in speech performance tests, Pearson’s correlation test or a Spearman rank correlation test will be performed. Electrode impedance values will be compared between the groups with the unpaired *t*-test (or the Mann-Whitney *U* test). CVC word score tests (with and without noise) will be compared between the groups with the unpaired *t*-test. Within-group comparisons will be calculated with differences of mean values. A clinically relevant speech performance decrease is a speech performance test score decrease of ≥ 7% when scoring between 30 and 80%, and ≥ 5% when the patients’ scores < 30 or > 80% on the speech performance test, a definition based on clinical experts. Incidence and degree of complications will be reported by means of frequencies.

Participants who withdraw from the study prematurely will be considered lost and will be replaced. Reasons for withdrawal or premature termination will be documented. Potential missing data will be handled using multiple imputation. Complete case analyses will be performed as a sensitivity analysis. All analyses will be performed on an intention-to-treat basis.

### Oversight and monitoring

#### Composition of the coordinating center and trial steering committee

Dr. H.G.X.M Thomeer (principal investigator) and Dr. L.M. Markodimitraki (research physician)

- Design and conduct of the COMFIT trial
- Preparation of protocol and revisions
- Preparation of case report forms
- Organizing steering committee meetings
- Identification of potential recruits
- Taking informed consent
- Supervising the trial
- Bi-weekly meetings
- Members of Trial management committee

Trial management committee

- (see title page for members)
- Agreement of final protocol
- Reviewing conduct and progress of study and if necessary agreeing changes to the protocol
- Advice on management matters
- Monthly meetings

#### Data management

Trial quality will be monitored independently by a local monitor (UMC Utrecht) once a year. The local monitor will check 10% of signed ICs, inclusion and exclusion criteria, source data, and serious adverse events (SAE). From the first three participants, the inclusion and exclusion criteria will also be checked. The study does not have a public involvement group [29].

### Harms

The investigator will submit a summary of the progress of the trial to the accredited MREC once a year. Information will be provided on the date of inclusion of the first participant, numbers of participants included and numbers of participants that have completed the trial, serious adverse events (SAEs)/serious adverse reactions, other problems, and amendments.

### Ethics and dissemination

The results (positive or negative) of this study will be disclosed unreservedly. Data and results of research are owned by the investigators. The results of the research will be submitted for publication in peer-reviewed scientific journals. Disputes on the interpretation of the results may not lead to an unnecessary delay in publication. None of the parties concerned has a right of veto. In addition, trial results will be communicated via symposia and relevant conferences on otology and cochlear implantation. Results will be summarized for the general public and interested trial participants and shared on the sponsor’s website.

The sponsor has an insurance which is in accordance with the legal requirements in the Netherlands (Article 7 WMO). This insurance provides cover for damage to research subjects through injury or death caused by the study. The insurance applies to the damage that becomes apparent during the study or within 4 years after the end of the study.

## Discussion

Cochlear implantation by means of creating a tight subperiosteal pocket without drilling a bony seat is a minimally invasive fixation technique with many advantages. However, the safety of this technique has not yet been proven with certainty. The objective of this study is the comparison of two broadly used surgical techniques for the fixation of the receiver/stimulator device during cochlear implantation, with and without drilling. This is the first randomized controlled trial that directly compares the minimally invasive technique with the conventional method of drilling a bony seat. Multiple outcomes will be assessed, using objective measures for the assessment of R/S device and electrode array migration, speech performance, and patient experience. A limitation of this trial is the monocenter design, which may affect recruitment rate and external validity.

## Trial status

Protocol version 2, 20-01-2022. The trial is currently in the recruitment phase. The first patient was recruited on 27 October 2021. Thirteen of 112 patients were included in the study on 11 May 2022. Approximate date of trial completion: 27-10-2023.

### Supplementary Information

**Additional file 1.**

## Data Availability

Only research members directly involved in this study and the monitor of the study will get access to all of the collected research data. Any data required to support the protocol can be supplied on request. Data sharing including the participant dataset, full protocol, and statistical codes will be considered upon reasonable request.

## Abbreviations

3D: Three-dimensional
AE: Adverse event
CBCT: Cone-beam computed tomography
CI: Cochlear implant
COMPASS: Cochlear implant awareness questionnaire
CVC: Consonant-vowel nucleus-consonant, Dutch speech perception test
ENT: Ear, nose and throat
IC: Informed consent
ICP: Iterative Closest Point
IRB: Institutional Review Board
MERC: Medical research ethics committee (MREC); in Dutch: medisch ethische toetsing commissie (METC)
R/S: Receiver/stimulator device
WMO: Medical Research Involving Human Participants Act; in Dutch: Wet Medisch-wetenschappelijk Onderzoek met Mensen
